# Comparative addition of dexmedetomidine and fentanyl to intrathecal bupivacaine in orthopedic procedure in lower limbs

**DOI:** 10.1186/s12871-018-0531-7

**Published:** 2018-06-06

**Authors:** Poupak Rahimzadeh, Seyed Hamid Reza Faiz, Farnad Imani, Pooya Derakhshan, Saeed Amniati

**Affiliations:** 1 Pain Research Center, Iran University of Medical Sciences, Tehran, Iran; 2 Rasoul-e-Akram Clinical Research Development Center, Iran University of Medical Sciences, Tehran, Iran; 3 Rasoul-e-Akram Medical Complex, Iran University of Medical Sciences, Tehran, Iran

**Keywords:** Lower limb surgery, Dexmedetomidine, Fentanyl, Intrathecal, Pain management

## Abstract

**Background:**

Spinal block is a common procedure for lower limbs surgery. Fentanyl, a synthetic opioid and dexmedetomidine, a selective α2 agonist have been used as adjuvants in spinal anesthesia to prolong intraoperative and postoperative analgesia. The aim of current study is to compare the efficacy of dexmedetomidine and fentanyl added to intrathecal bupivacaine in orthopedic procedures in lower limbs.

**Methods:**

In this randomized clinical trial, 90 patients undergoing elective lower limb surgeries were randomly allocated to three groups. Via intrathecal approach, the patients received 2.5 ml hyperbaric bupivacaine 0.5% plus 5 micrograms dexmedetomidine (BD group), 25 micrograms fentanyl (BF group) or 0.5 ml normal saline (BN group), respectively. Time to reach the complete motor block, the highest sensory level, regression from block, analgesic request and duration of the drug effect, hemodynamic changes and side effects were compared between the groups.

**Results:**

There was no significant difference between the groups regrading time to reach complete motor block, but time to reach the highest sensory level was shorter in group BD than group BF (6.28 ± 1.75 vs. 7.17 ± 1.45, *p* = 0.03). Group BD had significantly lower mean of NRS 6 h after operation (1.90 ± 0.84 vs. 6.16 ± 1.44 vs. 6.30 ± 1.17, *p* < 0.001) and longer duration to regress to Bromage 0 (331.60 ± 73.96 vs. 185.56 ± 35.87 vs. 147.03 ± 33.05 min, *p* < 0.001), to analgesic request (496.63 ± 70.19 vs. 296.33 ± 44.83 vs. 221.83 ± 22.26 min, *p* < 0.001), to regress two sensory levels (149.00 ± 23.17 vs. 88.90 ± 12.85 vs. 69.33 ± 6.67 min, *p* < 0.001) and to regress to S1 (560.53 ± 81.86 vs. 329.83 ± 44.10 vs. 241.83 ± 22.26 min, *p* < 0.001). Serial changes in SBP (*p* = 0.006), DBP (*p* = 0.03) and HR (*p* = 0.002) in group BF were significantly higher than the other two groups. The three groups had comparable side effects.

**Conclusions:**

Using dexmedetomidine as an adjuvant to bupivacaine for spinal anesthesia in lower limb surgeries has longer duration of sensory and motor block and longer postoperative analgesia.

**Trial registration:**

IRCT registration number: IRCT2017041010599N15, 24 May 2017.

## Background

Lower limb surgeries could be performed under local, neuroaxial and general anesthesia, but neuroaxial block is the preferred method. Spinal block has rapid onset, deep block, lower risk of infection and is cost effective. However, post-operative pain is an important problem as the used drugs have limited duration of effect; so the post-operative analgesic administration is necessary [1, 2].

Administrating the combinations of other classes of analgesics with local anesthetics has used to increase the duration and reduce side effects of analgesia [3]. Some drugs have been used as adjuvants in spinal anesthesia to prolong intraoperative and postoperative analgesia [1, 2] including opioids, α2 agonists, neostigmine, vasoconstrictors, etc. Clonidine and dexmedetomidine are two α2 agonists affecting via pre- and post-synaptic α2 receptors [4]. Dexmedetomidine has been widely used for anesthesia and analgesic purposes. This drug has sedative, anti-anxiety, analgesic, neuroprotective, and anesthetic-sparing effects [5]. Dexmedetomidine along with other drugs have been used to increase the duration of analgesia in subarachnoid, epidural and caudal blocks [6, 7].

Fentanyl is a synthetic opioid with central action, which is used widely for pain control. Intrathecal fentanyl is usually added to other local anesthetics to increase anesthesia and analgesia. It has improved spinal anesthesia and reduced the anesthetic drug related side effects including pruritus, nausea and vomiting [8].

Dexmedetomidine and fentanyl have been used as adjuvant to local anesthetics in different surgeries to provide superior analgesia and to improve the duration of the block [9–11]. One study on lower limbs surgery showed a better efficacy with dexmedetomidine [12].

In this study, we aim to compare the efficacy of dexmedetomidine and fentanyl added to intrathecal bupivacaine in orthopedic procedures in lower limbs in terms of block strength and time.

## Methods

In this randomized double-blinded clinical trial, patients between 20 and 65 years old, American Society of Anesthesiologist (ASA) grade I and II of either gender undergoing elective lower limb surgeries at Rasoul-e-Akram Hospital in 2017 were recruited. If the patients were addict and very obese, had uncontrolled hypertension or diabetes mellitus, renal or hepatic failure, cardiac block or dysrythmia, coagulopathies, neurologic disorders, hypersensitivity to any of the study drugs and known contra indications to spinal anesthesia, they would be excluded. The study was reviewed and approved by the Iran University of Medical Science Ethics Committee and written informed consent was obtained from all subjects before inclusion in the study.

The study sample size was calculated by $n=\frac{\left({s}_1^2+{s}_2^2\right){\left({z}_{1-\frac{\alpha }{2}}+{z}_{1-\beta}\right)}^2}{{\left({\overline{x}}_1-{\overline{x}}_2\right)}^2}$ using the results of Yektash et al.(2014) and through the following formula. Considering α = 0.05, power of 90% was calculated 29 per each group. Given that “g” is the number of group, then the total sample size was 82 by using the following formula: *n*^'^ = *n* ∗  √ (*g* − 1) for each group as there was possibility that some patients do not complete the study; 10% drop-out rate, we included 30 patients for each group [13, 14].

**Fig. 1:**
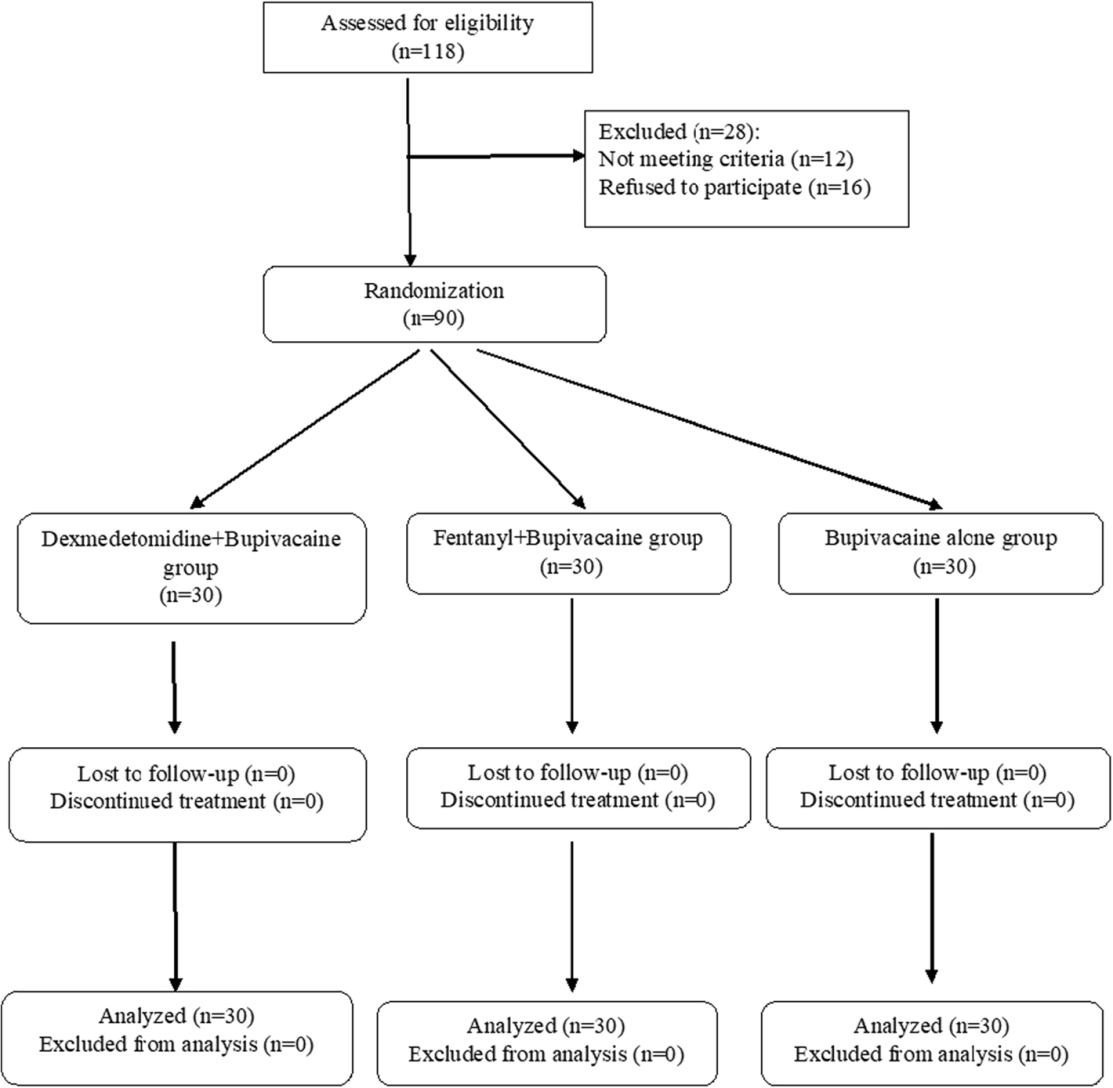
Flowdiagram of study protocol

Using Block Randomization, according to sample size, the patients were enrolled into the study (Fig. 1).

The patients were randomly allocated to bupivacaine (Marcaine spinal 0.5% heavy Astrazeneca, Cenexi, France) and normal saline (BN), bupivacaine and dexmedetomidine (Precedex, Hospira co., USA) (BD) and bupivacaine and fentanyl (Caspian Darou, Rasht, Iran) (BF). The patients received 2.5 ml intrathecal hyperbaric bupivacaine with 0.5 ml normal saline (BN) or 5 micrograms dexmedetomidine (BD) or 25 micrograms fentanyl (BF). All medications were prepared in 3 ml syringes. The patients and physician evaluating the outcome of the treatments were blinded to the group allocation.

All the patients were kept for 8 h fasting prior to surgery. Preloading completed with Ringer lactate solution (5 ml/kg body weight). Standard monitoring including noninvasive blood pressure (NIBP), ECG, heart rate and pulse oximetry performed. All patients received supplemental oxygen via mask (5 l/min). Under proper aseptic conditions, spinal anesthesia was given at the level of L4-L5 interspace in sitting position using a midline or paramedian approach by a 25G Quincke spinal needle. The anesthetic medication is injected at a rate of approximately 2 ml/sec; and then all patients were made supine.

Blood pressure, heart rate and pulse oximetry were performed every minute in the first 10 min and then every five minutes for one hour. We recorded systolic and diastolic blood pressure and heart rate before regional anesthesia and in the 5, 10, 15, 30, 45 and 60 min after anesthesia. All data were recorded in a data sheet specified to each patient.

As we needed anesthetic effects, both sensory and motor status were assessed prior to the spinal injection, then every 2 min after injection until reaching the highest sensory level and Bromage scale reaching to Bromage 3. After surgery, assessment performed every 10 min until the time to regression of 2 sensory levels, then every 20 min until the regression time to the dermatome S1 and motor scale to Bromage 0.

The motor dermatome level was assessed according to the Bromage scale:

Bromage 0 (none): Free movement of legs and feet.

Bromage I (Partial): Just able to flex knees with free movement of feet.

Bromage II (Almost complete): Unable to flex knees, but with free movement of feet.

Bromage III (Complete): Unable to move legs or feet.

Severity of pain 6 h after surgery was measured by Numeric Rating Scale (NRS). The patients were asked to rate their pain from a scale of 0 = no pain to 10 = the worst possible pain. In case of any side effects it was recorded.

Hypotension was defined as decrease in systolic blood pressure (SBP) more than 30% of baseline or SBP < 90 mmHg. If hypotension occurred, 10 mg ephedrine would be administered. Bradycardia was defined as heart rate (HR) below 50 pulses per minute and if occurred, 0.6 mg atropine would be administered.

### Statistical analysis

All data were analyzed using SPSS24 (version 24; SPSS Inc., Chicago, IL). The results are expressed as Mean ± standard deviation or percentage. The nominal categorical data between study groups were compared by using the chi-squared test or Fisher’s exact test as appropriate. One-way ANOVA and repeated measure of ANOVA were used to evaluate the changes in the variables during the study period. *p-*values ​​of less than 0.05 were considered statistically significant.

## Results

Ninety patients were randomly allocated to three groups of 30 patients. There was no significant difference between the groups in baseline findings (Table 1).

**Table 1:** Baseline findings between groups

		BN group (*n* = 30)	BF group (*n* = 30)	BD group (*n* = 30)	*P* value
Age (years)		39.43 ± 14.82	39.26 ± 15.81	42.20 ± 15.32	0.7
Gender	Male	20 (66.7%)	21 (70%)	19 (63.3%)	0.86
	Female	10 (33.3%)	9 (30%)	11 (36.7%)	
Weight (kg)		69.90 ± 13.45	73.90 ± 11.44	72.40 ± 11.68	0.44
Height (cm)		171.66 ± 7.94	174.06 ± 8.26	171.83 ± 6.21	0.39
Body mass index (kg/m^2^)		23.65 ± 3.94	24.30 ± 2.79	24.48 ± 3.47	0.62

Characteristics of block between the three groups are demonstrated in Table 2. There was significant differences between BD with BF and BN groups in regression to Bromage 0 (*p* < 0.001), two segmental regression (*p* < 0.001), sensory regression to S1 (*p* < 0.001), time to rescue analgesia (*p* < 0.001) and NRS 6 h after surgery (*p* < 0.001) and between BF and BN in regression to Bromage 0 (*p* = 0.004), sensory regression to S1 (*p* < 0.001), time to rescue analgesia (*p* < 0.001), two segmental regression (*p* < 0.001), and between BD and BF in the highest sensory level (*p* = 0.03), but there was no significantly difference between groups in onset of Bromage 3 (*p* > 0.05).

**Table 2:** Characteristics of block between three groups

	BN group (*n* = 30)	BF group (*n* = 30)	BD group (*n* = 30)	*P* value
Time from injection to highest sensory level (min)	6.45 ± 1.67	7.17 ± 1.45	6.28 ± 1.75	0.08
Time of two segment regression from the highest sensory level (min)	69.33 ± 6.67	88.90 ± 12.85	149.00 ± 23.17	< 0.001
Time for sensory regression to S1 from highest sensory level (min)	241.83 ± 22.26	329.83 ± 44.10	560.53 ± 81.86	< 0.001
Onset to Bromage 3 (min)	5.55 ± 167	5.05 ± 1.81	4.80 ± 1.74	0.24
Regression to Bromage 0 (min)	147.03 ± 33.05	185.56 ± 35.87	331.60 ± 73.96	< 0.001
Time to rescue analgesia (min)	221.83 ± 22.26	296.33 ± 44.83	496.63 ± 70.19	< 0.001
NRS six hours after surgery	6.30 ± 1.17	6.16 ± 1.44	1.90 ± 0.84	< 0.001

In all three groups, the highest sensory block occurred in T6 dermatome (Table 3). T5 dermatome was the second highest in BD and BF groups, but T7 dermatome was the second highest block in BN group.

**Table 3:** Highest dermatome level of sensory block

	BN group (*n* = 30)	BF group (*n* = 30)	BD group (*n* = 30)
T4	0	1 (3.3%)	3 (10%)
T5	3 (10%)1	7 (23.3%)	8 (26.7%)
T6	12 (40%)	16 (53.3%)	12 (40%)
T7	10 (33.3%)	3 (10%)	4 (13.3%)
T8	5 (16.7%)	2 (6.7%)	3 (6.7%)

**Fig. 2:**
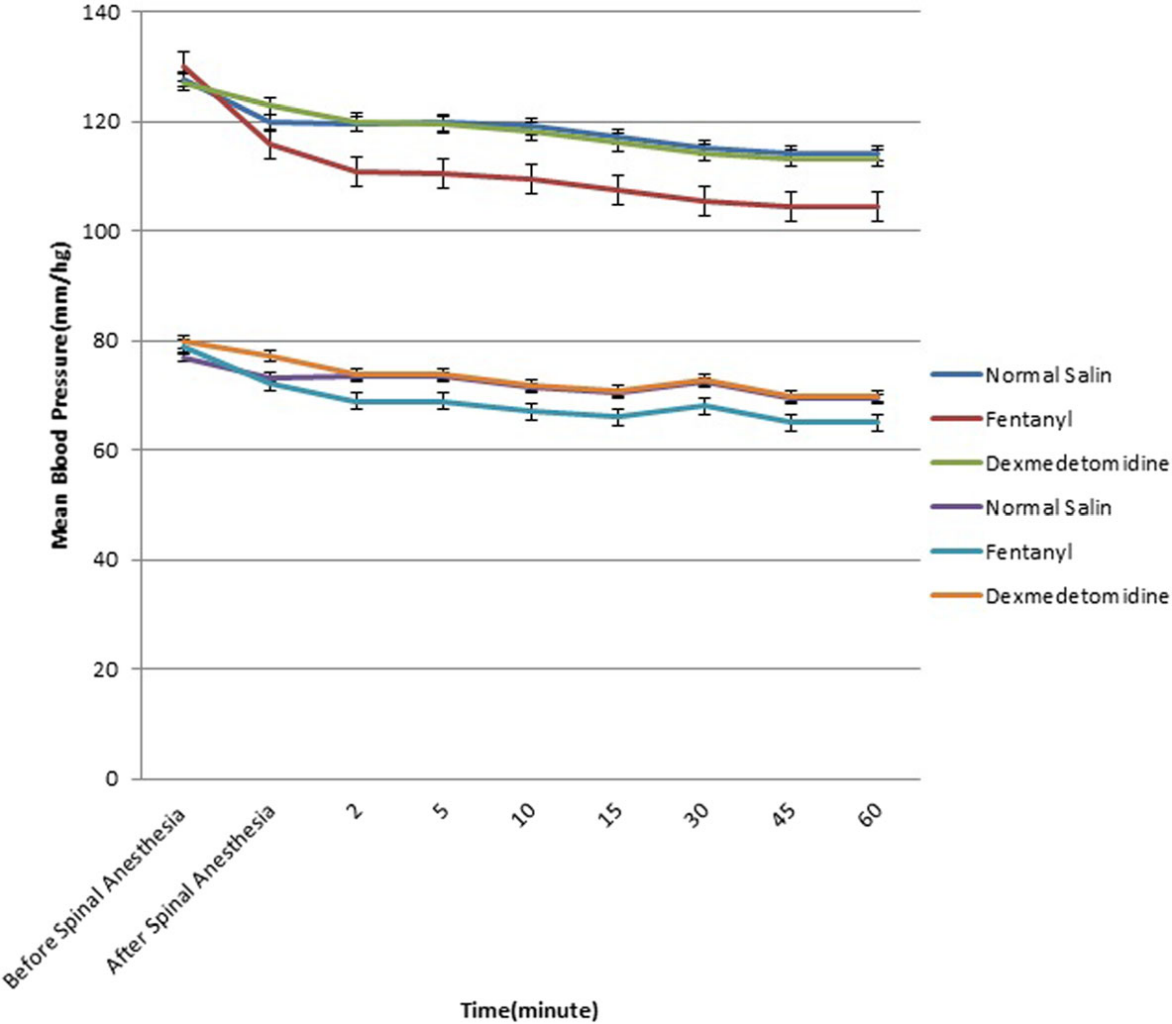
Error Bars of Systolic and diastolic blood pressure changes between groups before spinal anesthesia till 60 min after in between groups

**Fig. 3:**
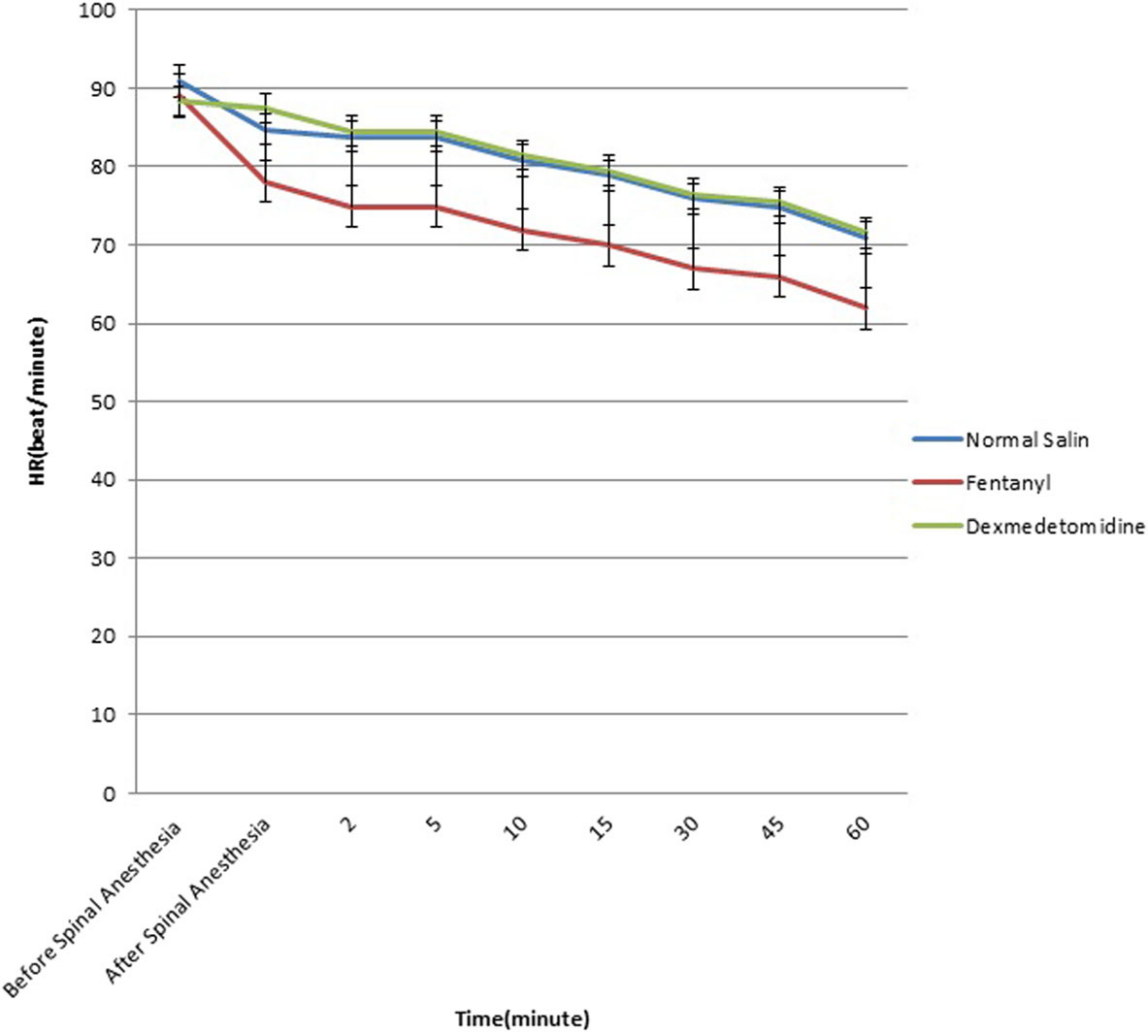
Error Bar of Heart rate changes between groups before spinal anesthesia till 60 min after in between groups

Figures 2 and 3 demonstrate Error bars of systolic and diastolic blood pressure and heart rate changes between groups before spinal anesthesia till 60 min after in each and between groups. Serial changes in SBP, DBP and HR were significant in each group (*p* < 0.001); these changes and reduction in SBP (*p* = 0.006), DBP (*p* = 0.03) and HR (*p* = 0.002) in BF group were significantly higher than BD and BN groups.

Nausea, vomiting and chilling were higher in BN group and hypotension and bradycardia were higher in BF group, but there were no significant differences between three groups regarding the treatment side effects (Table 4).

**Table 4:** Side effects between groups

	BN group (*n* = 30)	BF group (*n* = 30)	BD group (*n* = 30)
Nausea	2 (6.7%)	1 (3.3%)	1 (3.3%)
Vomiting	2 (6.7%)	0	0
Chilling	2 (6.7%)	2 (6.7%)	1 (3.3%)
Pruritus	0	2 (6.7%)	0
Hypotension	0	3 (10%)	1 (3.3%)
Bradycardia	0	3 (10%)	1 (3.3%)

## Discussion

In this study, we evaluated the efficacy of three spinal anesthesia methods, bupivacaine alone or with dexmedetomidine or fentanyl in lower limb orthopedic surgeries. Although there was no significant difference between groups in time to onset of Bromage 3 and complete motor block, BD group had lower time to reach the highest sensory level than BF group, with no difference with BN group but it was not statistically significant. (*P*-value = 0.08).

Similarly, Mahendru et al. [12] found no significant difference in onset of motor block between dexmedetomidine and fentanyl groups. While Yektas [13] and Ravipati [15] reported faster onset of motor block for dexmedetomidine compared to fentanyl. Other studies have also mentioned lower time to reach the highest sensory level in dexmedetomidine compared to fentanyl [12–19].

The mechanism of how dexmedetomidine prolongs sensory and motor blockade is not known. Dexmedetomidine is a highly-selective α2-adrenergic receptor agonist that causes analgesia by suppression the release of C fiber transmitters and hyperpolarization of post-synaptic neurons [13].

In our study, the highest sensory level in BD and BF group were T6 and T5 while in BN group was T6 and T7 dermatomes. One study reported the highest sensory level at T5 dermatome [15] and Mahendru [12] reported in T6 dermatome. Other study reported the highest sensory level at T5 dermatome in dexmedetomidine and T6 in fentanyl group [20].

None of the patients requested analgesic during the surgery. Bromage 3 occurred in all patients before operation. Complete regression of motor block (Bromage 0) was reached in all patients and with the highest duration in BD group. Moreover, time to regression to S1 sensory level and regression of two sensory levels in BD group was significantly longer than the other groups. These patients also experienced lower pain intensity six hours after surgery indicative of the highest postoperative analgesia duration in BD group.

Reduced need for analgesics in the post-operation period, more stable hemodynamics, longer duration of sensory and motor block for dexmedetomidine have been reported in previous studies comparing this drug with other drugs such as clonidine, fentanyl and sufentanil [16, 19, 21–24]. In orthopedic surgeries of lower limb, better results have also been reported for dexmedetomidine compared to fentanyl [12, 13].

Hemodynamic changes is common in anesthesia medications. We observed that changes in SBP, DBP and HR in BF was higher than BD and BN groups, with no difference between BD and BN patients. The highest decline occurred 5 min after spinal injection and was rather stable afterwards. Unlike our findings, other studies did not report any significant difference between fentanyl and dexmedetomidine regarding hemodynamic status [12, 13, 15–18]. Decline in HR and blood pressure are common effects of opioids. The difference in hemodynamic findings could be due to the response of each individual to the drug, demographic profile, volume of IT injectate and volume of diluent used.

Side effects may occur by using any anesthesia medications. The best medication is the one with the highest efficacy and lowest side effects. We observed no significant difference in the rate of hypotension, bradycardia, nausea and vomiting and chilling between groups. Previous studies have reported different rate of side effects. Similar to our findings, Ravipati [15] observed pruritus only in fentanyl group while nausea and vomiting was more common in dexmedetomidine, with no significant difference between groups. There is also only one study reporting increase in hemodynamic side effects, bradycardia and hypotension, in dexmedetomidine [24].

Another important side effect of anesthesia medications is respiratory system suppression. However, we observed no respiratory suppression. First, fentanyl compared to other opioids is less likely to cause respiratory suppression. Second, this complication is not common in dexmedetomidine.

In order to reach better efficacy, we can increase the dose of the used dexmedetomidine. Gupta [19] reported that increasing the dose of dexmedetomidine from 2.5 μg to 10 μg would show better and longer sensory and motor block, with longer duration of anesthesia and comparable hemodynamic and side effects profile.

## Conclusion

In conclusion, using dexmedetomidine as an adjuvant to bupivacaine for intrathecal analgesia in lower limb surgeries has longer duration of sensory and motor block, longer postoperative analgesia with low side effects. All three treatments had lower side effects with no difference between groups.

## Abbreviations

ANOVA: Analysis of variance
ASA: American Society of Anesthesiologist
BD: Bupivacaine + Dexmedetomidine
BF: Bupivacaine + fentanyl
BN: Bupivacaine + normal saline
ECG: Electrocardiogram
HR: Heart rate
NIBP: Noninvasive blood pressure
NRS: Numeric Rating Scale
SBP: Systolic blood pressure
SPSS: Statistical Package for the Social Sciences
